# Financial performance of the teaching pharmacies in Isfahan: an economic evaluation

**Published:** 2009

**Authors:** A.M. Sabzghabaee, M. Etebari, H. Sajjadi, Sh. Badri, S.M. Hosseini-Biuki, R. Sheikhaboumasoudi

**Affiliations:** 1 *Clinical Toxicology Research Center, Isfahan University of Medical Sciences, Isfahan, I.R.Iran*; 2 *Department of R&D, Vice-chancellery for Administrative and Financial Affairs, Isfahan University of Medical Sciences, Isfahan, I.R.Iran*; 3 *Department of Pharmacotherapy, Tehran University of Medical Sciences, Tehran, I.R.Iran*; 4 Department of Healthcare Management, School of Management and Medical Bioinfomatics, Isfahan University of Medical Sciences, Isfahan, I.R.Iran

**Keywords:** Economic evaluation, Teaching pharmacy, Cost, Income, Isfahan

## Abstract

Teaching pharmacies are amongst the important cornerstones of a healthcare system for drug supplying, pharmacy education and pharmacy practice research. Assessment of the Iranian healthcare system costs shows that after personnel charges, drug outlay is the second expensive factor. This great financial mass requires integral audit and management in order to provide costumers satisfaction in addition to financial viability. Teaching pharmacies are required to realize financial viability as well as providing several educational and drug servicing goals, which makes microeconomic analysis important. The aim of this study was to evaluate the financial performance of the teaching pharmacies affiliated with the Isfahan University of Medical Sciences (with the *abrreviated names as*: SHM, ISJ, AZH *for the confidentialiy of the financial data*). This is a descriptive and cross-sectional study done in 2008. The target pharmacies of this study were all the 3 teaching pharmacies affiliated with the Isfahan University of Medical Sciences. The data collecting template was prepared using the standard scientific methods according to the goals of this research The goals also nominated necessary items needed in economic profit evaluation. The data collection template was completed by reference to the teaching pharmacies financial documents and reports, used as a base for calculating the total income and the total costs in 2007-2008 financial year. The difference between these two balances showed the value of profits or loss. The profit/cost ratio was also calculated, using the proportion of the total income to the total costs. The collected data was statistically analyzed using the Excel software (Microsoft 2007). For the financial year 2007-2008, the difference between the total income and the total costs was -831.6 million Rials (excess costs to income) for the SHM pharmacy, + 25.4 billion Rials for the ISJ pharmacy and -429.5 million Rials for the AZH pharmacy. According to our findings there is a strong requirement to improve the financial performance of all the three teaching pharmacies while maintaining a high standardard of teaching and educational affairs.

## INTRODUCTION

Health economics is a new branch of economic science, started in the early 1950s when health care system costs were noticed as a general basic need and therapeutic services became an industry (1). The systematic use of economic theories in healthcare system fields started at 1970s (2). Health economics is defined as the study of quality, costs and value of constricted wealth specified for health care purposes and therapeutic aims (3). Evaluating methods which mix limited resources to provide efficient and standard health-care services is another target for health economic surveys. This methodology is the key tools for assessing the financial efficiency of health care facilities (4). Stark assessment of economic properties related to health care system which is considered as a philanthropic act looks ineffectual but the studies based on appraising the services efficiency and rational utilization of resources may be considered salutary (5). Economic evaluation in health care systems has many research aspects including: cost-benefit, cost-utility, cost-effectiveness, etc. In these type of studies, all direct and indirect costs and expenditures of an independent variable e.g. drug therapy, disease or any especial health care service is determined first and is analyzed versus its benefit, utility or effectiveness (2).

To the best of our knowledge, a limited number of studies have been performed and published that economically evaluate critical foci of the Iranian health care system including pharmacies (6–12). Towfighi and colleagues assessed the profit/cost ratio in a teaching heart hospital pharmacy in Tehran as the first one of its kind in Iran (7). In 2001, the department of drugs and narcotics regulation of the Iranian ministry of health has assessed the economic properties of a sample cohort of Iranian community pharmacies (6). In addition, some studies assessed the economic performance of other clinical or para-clinical departments for example the study conducted by Mohammdi and colleagues which analyzed economic performance of the Shahid Madani hospital laboratories in Khoramabad in 2000 financial year (8)or the one conducted by Ayatollahi and colleagues which evaluated the profit/cost ratio in insurance systems of Fars province (9). Heidari, Mortazavi, and Gheisari carried out studies evaluating hospitals financial performance which are still on the start point of the long economic evaluation pathway (10–12).

Nowadays, the ever increasing demand of patients for obtaining information about their physician-prescribed drugs create new expectations from community pharmacists. Pharmacist’s high potentiality for delivering clinical information and their communication skills at the time of facing patients seem to help the drugstore managers to improve their financial profitability. The increasing demand of the pharmacy clients and patients who are seeking their needed specialized information about rational use of drugs made this ring of health chain much more important than it used to be. Most of the faculties of pharmacy in Iran, own at least one or more not-for-profit teaching pharmacy for the purpose of teaching and simulating the real situation of pharmaceutical care provision. These drugstores are known by the people as the governmental pharmacies. People generally believe that these drugstores are more trustable and they offer some drugs that are not ordinary found in other pharmacies and this fact has an undeniable direct positive effect on their financial turn over and economic balance.

The importance of financial performance and its direct effects on income, teaching and service provision level in educational pharmacies, made good reasons for the design and conduction of present study which evaluates the above mentioned objectives for the teaching pharmacies affiliated with Isfahan University of Medical Sciences (IUMS). The results of present study are hoped to be useful for future management goals and the pharmaceutical policymakers and authorities.

## MATERIALS AND METHODS

The research protocol and methodology (including the final data collection template and permission to access the confidential financial data) of this study as well as publication of the data was approved by the higher research committee of the School of Pharmacy and Pharmaceutical Sciences of the IUMS. This descriptive and cross-sectional study was conducted in 2008. The target pharmacies included the three teaching community pharmacies owned by the above mentioned school of pharmacy. These out-patient pharmacies named SHM, ISJ and ALZ (*abbreviated for the confidentiality of the financial data*), are located in three different geographical area of the city and each is next to a teaching medical center again affiliated with IUMS and the pharmaceutical services are open to public. SHM and ISJ pharmacies are the two most important drug servicing centers for special cases of drug supply and needed drugs in Isfahan province.

In order to prepare the evaluation data collection template, the goals and objectives of the study were initially reviewed by all authors carefully and the nominated necessary items needed in this financial and economic assessments were determined (13). Then the data collection template was prepared according to the method described by Schumacher (14)and modified by Miller (1). The template was further discussed and adapted for usage in Iran by a panel of experts including the relevant administrative, scientific and technical experts (15). The final template is summarized in the next section (Table 1). Before the start of the field study, the administrative managers and personnel of the finance departments for all three pharmacies had an educational meeting and the goals and objectives of the study were explained to them by one of the authors (AMS). The data collection templates were completed and done directly by two of authors (HSS & SHB) by reference to the teaching pharmacies official financial documents and reports for the financial year 2007-2008 (known as a base to calculate total costs and income in 2007 financial year). The 2007-2008 financial year was chosen because of the accessibility of up-to-date financial data. The Difference between the total income and total cost shows the value of profits or loss.. The collected data was statistically analyzed using Microsoft Excel^®^ (2003) software and commented according to the study aims.

**Table 1:** Economic performance of all three teaching community pharmacies affiliated with Isfahan School of Pharmacy and Pharmaceutical Sciences in 2007-2008 financial year.

	SHM	ISJ	ALZ
Total number of personnel	16	31	17
Total personnel cost*	1027.7*	1776.7	1016.5
Capitation for each employee	64.2	57.3	59.8
Total consumption ingredients costs	7.9	64.6	7.5
Energy costs	2.9	11.8	2.9
Connection costs	1.2	5	0.5
Total depreciation¤	9.1	1269.4	140.6
Total income	589.7	29044.1	1019.1
Total indirect income (Non-Functional)	0 (0)#	0 (0)	0 (0)
Total direct income (Functional)	589.7 (100)	29044.1 (100)	1019.1 (100)
Prescription drugs sale portion	8414.3 (97)	71658.4 (98,9)	DNA†
Compounding drugs sale portion	6.3 (0.1)	192.9 (0.1)	DNA†
Cosmetic-Hygienic paraphernalia sale portion	338.5 (2.9)	574.2 (0.8)	DNA†
Total cost	1,421.3	3,647.8	1,448.7
Total indirect costs§ (Non-functional)	369.8 (26)	524 (14.2)	278 (19.2)
Total direct costs (Functional)	1051.6 (74)	3132.9 (85.8)	1170.8 (80.8)
Difference between total income and costs income	−831.6	+25,396.3	−429.6

* Including patron parts allotted shares

^**^All figures are in million Rials.

¤ Including pharmacy structure depreciation, permanent wealth and vehicles

# Figures in brackets represent the percent of corresponding cost/income

§ Including teaching, rent, food, distribution, sale and other miscellaneous costs

† DNA Data Not Available, −ve vlaues show a loss.

## RESULTS

The calculated financial indices of the pharmacies studied are summarized in Table 1. It should be noticed that the number of employees of the three pharmacies were not equal (Table 1). Fixed financial ratios for evaluating the economic performance of pharmacies were calculated and presented in Table 2. Both tables are fully discussed on the next section.

**Table 2:** Fixed financial ratios of all three teaching community pharmacies affiliated with Isfahan School of Pharmacy and Pharmaceutical Sciences in 2007-2008 financial year.

	SHM	ISJ	ALZ
Functional income/ Functional costs	0.56	9.30	0.87
Functional income/ Functional costs (Depreciation excluded)	0.57	15.6	0.99
Non-functional income/ Non-functional costs	0.00	0.00	0.00
Total income/ Total costs	0.41	7.90	0.70
Total income/ Total cost (Depreciation excluded)	0.42	12.2	0.78
Total income/ Functional costs	0.56	9.30	0.87

## DISCUSSION

As an introduction to this section, it should be mentioned that because of the lack of similar published studies on financial evalu-ation of teaching (out-patient) pharmacies and novelty of this issue in Iran, the results and ratios derived from them could not be discussed by comparing to other studies. Therefore, we have discussed the results with regard to the differences between the three teaching pharmacies as below.

According to our findings the functional income/functional costs ratio for the SHM pharmacy was 56% (Table 2). This ratio showed a 461.8 million Rials loss, which may be caused by the excess of costs to incomes. Calculating net loss requires deducting pharmacy permanent wealth depreciation as temporary costs. The functional income/ functional costs ratio after excluding depritiation cost (table 2) showed slight improvement compared with the first one. This fact emphasizes that making the pharmacy financially independent may cause a crisis which may end with being bankrupt. The total income/total costs, as the third ratio is 41%. Economic evaluation of the SHM pharmacy showed that its total income can offset only 41% of its costs. This is possibluy due to a 369.8 million Rials non-functional cost against zero non-functional income, redounding on worse situation compared with the first and the second ratio (Table 1). Furthermore, low permanent wealth depreciation expenses in this pharmacy induced little improvement (about 1%) between the third and the forth ratio. Mentioning the third ratio as a financial indicator calculated from total costs and income seems necessary. The noticable difference between this figure and figure one shows a necessity for efficient manners which will result in increments.

The ISJ pharmacy with a substantial functional income of 29 billion Rials value (Table 1) had the ability to overlay the permanent and extra costs for 9.3 times (Table 2). This has led to a suitable profit in 2007--2008 financial year for this teaching pharmacy. Conversely, this drugstore has a depreciation expense more than 1.27 billion Rials (Table 1). This amount of money comprises 41% of the total functional overheads. This situation gave a functional income/functional costs ratio of 15.6% (excluding depreciation expenses) for this pharmacy. This drugstore had no functional income and thus yielded a non-functional/ functional income ratio of 0%. At the same time, the pharmacy had non-functional costs totalling 523.98 million Rials. Looking carefully at the forth ratio of Table 2 shows that the pharmacy’s total income can cover its total costs (functional and non-functional) 8 times over. This helps to ignore the third ratio but it’seems obvious that reducing non-functional costs, while there is no non-functional income, becomes necessary in the future. The fifth ratio is that of total income/total costs while depreciation costs is excluded,. Absence of the non-functional income affected the sixth ratio as well and, showed that the total income of this pharmacy can definitely overlay its functional expenses.

Economical evaluation of ALZ pharmacy in the same way showed the ratio of functional income/functional costs equal to 87% (Table 2). This shows that the pharmacy’s income could not overlay its expenses and left a 13% deficit. It also means a loss of more than 151.6 million Rials in 2007-2008 financial year for the drugstore. The proportion of detriment in this pharmacy compared to SHM pharmacy is much lower and it is also less than its own functional income. The true detriment of this pharmacy maybe better determined by discounting the permanent wealth depreciation from the calculated loss. However It seems that these expenses are not fully and completely documented, as is the case with the SHM pharmacy. In this state of affairs the second ratio emphasizes on a smaller loss in which income and costs are approximately (by 99%) equal. In other words, if the pharmacy was to be left to be managed on its own there would be no profit for the pharmacy and no loss either. High rate of non-functional costs and gaining no non-functional income caused the forth ratio to be equal to 70%. The raw figure shows that the total income of this pharmacy can only overlay 70% of its total costs. There incurs a 277.98 million Rials non-functional costs while there is no non-functional income. Therefore the balance deficit for the SHM pharmacy is 30% which shows a better condition compared with that of the SHM pharmacy, which shows a 59% balance deficit (Table 2). These figures demonstrate that it is essential that endeavors are made in order to reduce costs and increase the income to achieve financial viability of these two pharmacies.

## CONCLUSION

Comparison of the mentioned sextet ratios in SHM pharmacy, ALZ drugstore and ISJ pharmacy revealed that the ISJ pharmacy not only has the highest income but also can cover its expenses by its income. This evidence is sufficient for representing the efficiency in enterprising. Considering the forth ratio (total income/total costs), used to compare the organization financial properties with other organizations, helps to rank the three pharmacies economic performance as following:

ISJ Pharmacy >ALZ Pharmacy >SHM Pharmacy

The authors believe that similar economic evaluation is necessary as an important decision making element in other similar units.

In support of improving the financial performance of above mentioned teaching pharmacies (especially ALZ and SHM pharmacies), according to our literature survey, analysis of findings and discussions with relevant experts, these solutions are offered which maybe regarded as topics for further study:

- Study the effects of functional emoluments intensification.
- Designing and performing plans for a more efficient role in the drug market.
- Search for means to increase the non-functional revenue and its availability assessment.
- The use of value engineering technique to help in avoiding unnecessary expenses and probable repeats.
- Strategic planning for effective human resources management of these pharmacies
- Recognizing wastage of drug expenditure.
- Using efficient supervision ways to control pharmacy disbursements.
